# Phospholipase D2 Is Involved in the Formation of Golgi Tubules and ArfGAP1 Recruitment

**DOI:** 10.1371/journal.pone.0111685

**Published:** 2014-10-29

**Authors:** Narcisa Martínez-Martínez, Emma Martínez-Alonso, José Ballesta, José A. Martínez-Menárguez

**Affiliations:** Department of Cell Biology and Histology, Medical School, IMIB-Arrixaca, University of Murcia, Murcia, Spain; National Cancer Institute, United States of America

## Abstract

Lipids and lipid-modifying enzymes play a key role in the biogenesis, maintenance and fission of transport carriers in the secretory and endocytic pathways. In the present study we demonstrate that phosphatidic acid generated by phospholipase D2 (PLD2) is involved in the formation of Golgi tubules. The main evidence to support this is: 1) inhibitors of phosphatidic acid formation and PLD2 depletion inhibit the formation of tubules containing resident enzymes and regulators of intra-Golgi transport in a low temperature (15°C) model of Golgi tubulation but do not affect brefeldin A-induced tubules, 2) inhibition of PLD2 enzymatic activity and PLD2 depletion in cells cultured under physiological conditions (37°C) induce the formation of tubules specifically containing Golgi matrix proteins, and, 3) over-expression of PLD2 induces the formation of a tubular network. In addition, it was found that the generation of this lipid by the isoenzyme is necessary for ArfGAP1 recruitment to Golgi membranes. These results suggest that both proteins are involved in the molecular mechanisms which drive the formation of different types of Golgi tubules.

## Introduction

Membrane flux into, through and out of the Golgi complex is mediated by vesicular and tubular transport intermediates. Much information is available on the composition and the molecular machinery involved in the formation and fusion of COPI-, COPII- and clathrin-coated vesicles with the corresponding target membranes; for example, it is known that coat complexes, tethering factors and Rab and SNARE proteins play a key role in these processes [1]–[3]. More recently, lipids and lipid-modifying enzymes have been added to this list of important regulatory molecules that are necessary for the deformation of the membrane during budding and fission. In contrast, despite the fact that they are a common feature of the secretory (and endocytic) pathway, little is known about the mechanisms regulating tubular transport intermediates [1]–[3].

The first indications about the molecular machinery involved in the formation of tubules came from studies on brefeldin A (BFA), a fungal drug that induces extensive tubulation of the Golgi followed by fusion of the Golgi complex into the endoplasmic reticulum [4]. Analysis of its action showed that BFA induces the detachment of the COPI coats from membranes, and that the main target of this drug is GBF1, a GTP exchange factor (GEF) for Arf1 [5]. This interaction prevents activation of this small GTPase and the subsequent formation of COPI vesicles. BFA-induced Golgi tubules may be representative of the tubules that mediate COPI-independent Golgi-to-ER transport.

Another experimental condition that induces extensive Golgi tubulation is low temperature (15°C). In contrast to BFA-induced tubules, such tubules are enriched in some molecules (Golgi resident enzymes) but not others (anterograde and retrograde cargo, matrix proteins) [6]. Further analysis demonstrated the presence of specific Rabs and SNAREs in these tubules that are involved in intra-Golgi transport but not in ER-Golgi traffic [7]. Thus, low temperature-induced tubules may represent transport carriers operating in intra-Golgi transport, more specifically in the recycling of resident enzymes. A detailed explanation of the physiological significance of low-temperature-induced tubules and their putative roles in the framework of intra-Golgi transport models can be found in our recent review [8]. As described for BFA-induced tubules, the formation of low temperature-induced tubules may also depend on COPI machinery.

Tubule formation needs the deformation and further elongation of Golgi membranes, processes which may require a specific lipid composition [9]. There is growing evidence that glycerolipids, such as lysophosphatidic acid (LPA), phosphatidic acid (PA) and diacylglycerol (DAG) play an important role in tubule formation by mediating protein recruitment to membranes, by modulating protein functions or by directly affecting membrane curvature [10]–[12] Hence, the enzymes associated with the metabolism of these lipids, such as phospholipases, acyltransferases and lipid kinases, probably play a key role in tubule formation. It is known, for example, that the LPA generated by the enzyme phospholipase A_2_ is involved in tubule-mediated retrograde trafficking from the Golgi to the endoplasmic reticulum [13], [14]. Studies using the drug propranolol indicated that DAG is involved in the formation and/or fission of vesicles and tubules [15], [16]. Furthermore, recent studies indicate that lipid phosphate phosphatase 3, which generates DAG from PA, is also involved in tubule-mediated retrograde transport [17]. PA generated by phospholipase D2 (PLD2), in cooperation with the protein BARS, is involved in the fission of Golgi carriers [18]. The coordinate action of lipid modifying enzymes, LPA acyltransferase and phospholipase A_2_ in the biogenesis of vesicles and tubules has also been demonstrated [19]. Interestingly, these enzymes promote or inhibit COPI fission, respectively. The association with membranes of components of the COPI machinery depends on a specific lipid composition, as is the case with Arf [20] and ArfGAP1 [16], [21], [22].

The aim of the present study was to deepen our understanding of the molecular mechanisms that participate in tubule formation. More specifically, we analysed the role of glycerolipids and related enzymatic activities in the generation and/or maintenance of Golgi tubules as well as their relationship with the COPI machinery. Our findings reveal for the first time that PA generated via phospholipase D2 activity plays an active role in the formation of Golgi tubules and that it is involved in the recruitment of ArfGAP1 to the Golgi membranes.

## Results

### The formation of low temperature-induced tubules requires phosphatidic acid

**Figure 1:**
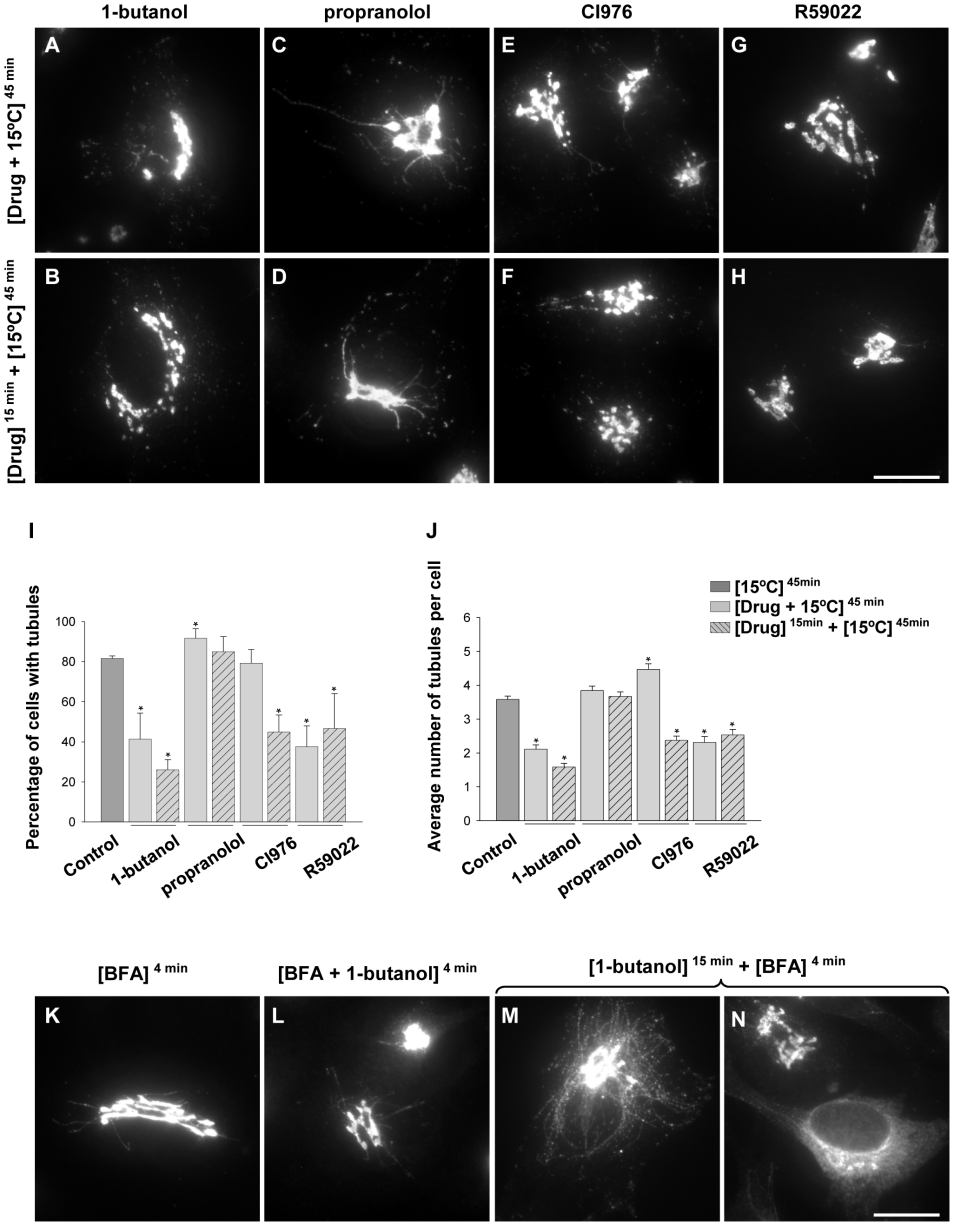
Effects of inhibitors in the formation of low temperature- and BFA-induced tubules. (A–H) Cells were incubated at 15°C for 45 minutes in the presence of inhibitor without (A, C, E, G) or with (B, D, F, H) pre-treatment with the same inhibitor for 15 min at 37°C and then immunolabelled for N-acetyl-galactosaminyltransferase T2 as Golgi marker. (I, J) Quantitative analysis. Bars represent the percentage of cells showing tubules (I) and the number of tubules per cell (J) obtained after each treatment (expressed as mean ± SEM). At least 300 cells were counted from three independent experiments. Asterisks indicate significant differences (Student t test p≤0.05). (K–N) Cells were incubated for 4 minutes with BFA in the presence of 1-butanol without (L) or with (M,N) pre-treatment with the same inhibitor for 15 min at 37°C and then immunolabelled for N-acetyl-galactosaminyltransferase T2 as Golgi marker. Bars, 20 µm.

As the initial model to ascertain the role of lipid and lipid-modifying enzymes in Golgi tubule formation, we used HeLa cells cultured at 15°C, a condition that stimulated the formation of tubules containing Golgi resident enzymes and SNARE and Rab proteins involved in intra-Golgi transport [6], [7]. Several enzyme inhibitors were used to characterise the lipid requirements for the formation of tubules in this model (Fig. 1). N-acetyl-galactosaminyltransferase T2 was selected as Golgi marker but results were confirmed with other resident proteins such as Rab6, Gos28 or galactosyltransferase. In the presence of 1-butanol, the hydrolysis of phosphatidylcholine by phospholipase D results in phosphatidylbutanol instead of PA, in a reaction known as transphosphatidylation [23]. To see how this primary alcohol affects the formation of low temperature-induced tubules, the number of cells with Golgi tubules was compared in cells cultured at 15°C for 45 min in the presence and absence of this compound. It was found that the number was halved in the presence of 1-butanol (Figs 1A, I), while many treated cells showed fragmented tubules throughout the cytoplasm (Fig. 1A). This effect was even more pronounced when cells were pre-treated with this compound for 15 min before cooling (Figs 1B, I). Additionally, our experiments showed that not only the number of tubulated cells was significantly reduced but also the mean number of tubules per cell (Fig. 1J). The concentration of alcohol used in this study (0.3% v/v) is efficient inducing transphosphatidylation [24] but did not alter Golgi morphology or cytoskeleton organization (data not shown). No significant inhibition of the tubulation was observed with 2-butanol, which is a poor substrate of PLD (data not shown). These results suggest that PA is necessary for the formation of this type of Golgi tubule. Unfortunately, it was not possible to use specific inhibitors of PLD1 and PLD2 in this model because they were seen to alter the Golgi structure when used in cell cultures at 15°C (data not shown).

Propranolol inhibits the formation of DAG through the dephosphorylation of PA, a reaction catalysed by phosphatidic acid phosphohydrolases/lipid phosphate phosphatases (PAP/LPP) [25]. In contrast with 1-butanol, this compound did not inhibit the formation of low temperature-induced tubules (Fig. 1C, D). In fact, there was a slight increase in the number of tubulated cells and the number of tubules per cell (Fig. 1I, J), suggesting that DAG is not necessary for the formation of this type of tubule. The increase, although small, in the number of tubules also pointed to a key role for PA.

PA can also be synthesised from LPA by the action of LPA acyltransferase, a reaction that can be inhibited by the pharmacological agent CI-976 [26], [27]. Treatment with this compound slightly reduced the number of cells with tubules but increased the mean number of tubules per cell (Fig. 1E, I, J), probably due to the accumulation of LPA [13], [14]. However, pre-treatment of the cells for 15 min with this compound before cooling reduced the number of tubulated cells and the mean number of tubules per cell by a half (Figs 1F, I, J).

The compound R59022 is an inhibitor of DAG kinase, which has the opposite effect to propranolol, inhibiting the formation of PA from DAG [28], [29]. As expected, it inhibited the formation of low temperature-induced tubules (Figs 1G, H–J). The results were similar with or without pre-treatment.

Previous studies using propranolol showed that DAG is necessary for the formation of BFA-induced Golgi tubules [16]. In agreement with these studies, we found that propranolol inhibited the formation of these tubules compared with the observations made in control conditions (data not shown). In contrast, 1-butanol increased the number of BFA-induced tubules (Fig. 1L–N). It was more evident when the cells were pre-treated with 1-butanol before the addition of BFA. Under this condition, 70% cells developed huge tubular networks (Fig. 1M). Interestingly, 30% of the cells showed a cytoplasmic labelling pattern after very short BFA treatment indicative that 1-butanol accelerated BFA-induced Golgi-endoplasmic reticulum fusion (Fig. 1N). These findings confirm that Golgi tubules induced by low temperature and BFA require different lipidic species.

### PLD2 is necessary for the formation of low temperature-induced tubules but not BFA-induced tubules

**Figure 2:**
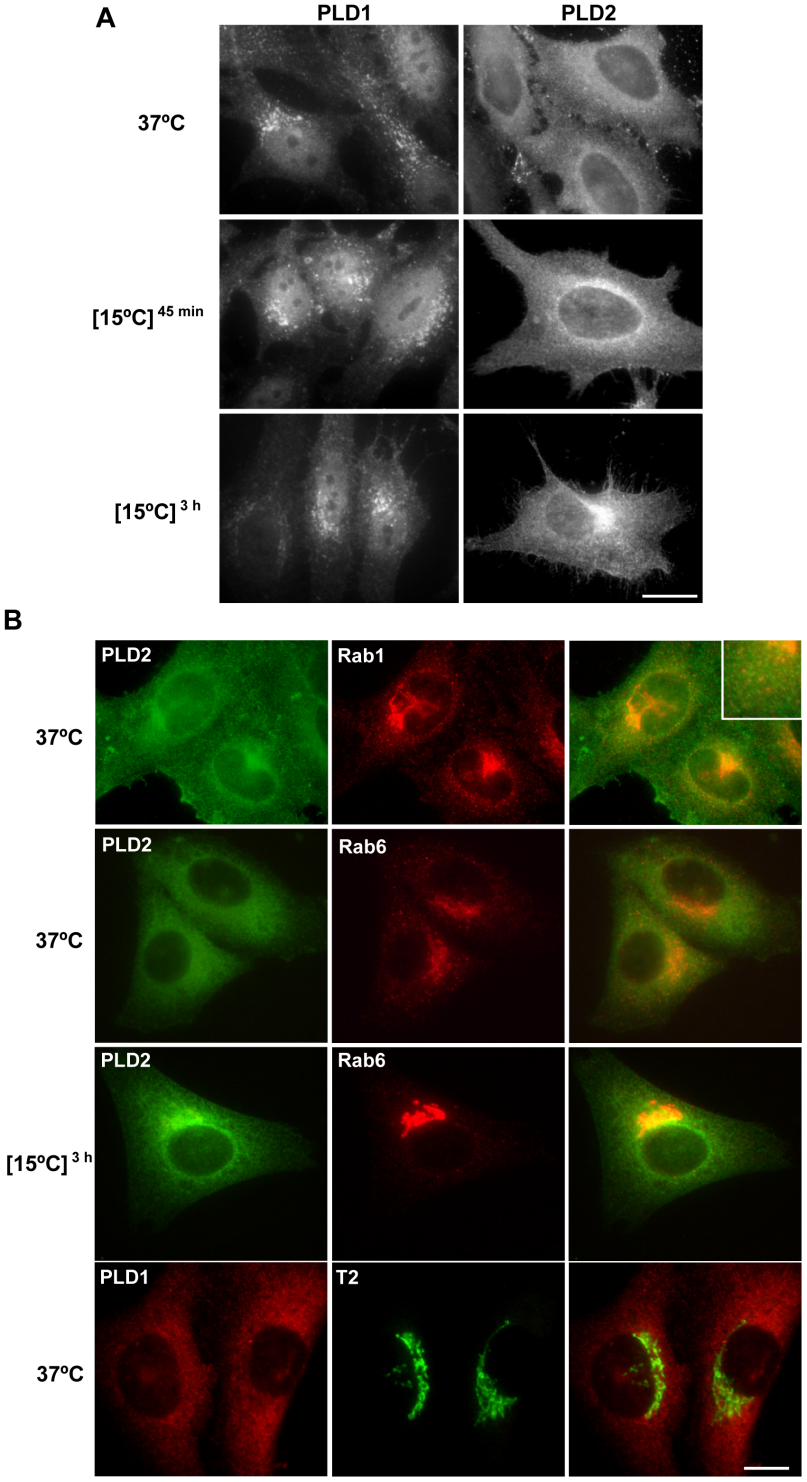
Effect of 15°C in the distribution of PLD1 and PLD2. Cells were cultured at 37°C, 15°C for 45 min or 3 h and immunolabelled for PLD1 and PLD2 (A) or double immunolabelled for PLD isoforms and Golgi markers (B). (A) In control and treated cells, PLD1 is located in small immunolabelled dots distributed throughout the cell. PLD2 showed a diffuse cytoplasmic labelling pattern. The incubation at low temperature concentrates PLD2 into the perinuclear area. (B) PLD2 but not PLD1 co-localizes with Golgi markers in the perinuclear area. PLD2 is also present in several Rab1-immunoreactive dots representing ERGIC elements. Bars, 20 µm.

The above results demonstrate that PA is required for low temperature-induced Golgi tubule formation. The most important route producing signalling PA uses phosphatidylcholine as source in a reaction catalysed by PLD [30]. The highest inhibition of tubule formation was obtained with 1-butanol, suggesting that this pathway is the most important for the formation and maintenance of this kind of tubule. In mammals there are two isoforms of this enzyme, called PLD1 and PLD2, both of which have been localised in the Golgi complex [31], [32]. It may be possible that one or both of these isoforms are involved in the formation of the PA necessary for Golgi tubule formation at low temperature. To explore this hypothesis, we first analysed whether low temperature affects the distribution of these isoforms. Under control conditions, PLD1 is found in spots distributed throughout the cytoplasm (Fig. 2A). In addition, it has also been observed in the nucleus [31]. No co-location with Golgi markers was observed (Fig. 2B). Low temperature did not alter its distribution. PLD2 showed a typical cytoplasmatic labelling pattern, which was more intense in the perinuclear area (Fig. 2A). Double immunolabelling experiments confirmed the Golgi location of PLD2 (Fig. 2B). Low temperature induced time-dependant concentration of PLD2 in the perinuclear area (Figs. 2A, B). In addition, PLD2 was also present in some Rab1-reactive spot distributed throughout the cell (Fig. 2B, inset), thus indicating the presence of this enzyme in ERGIC elements. Identical results were obtained with different anti-PLD2 antibodies. Since our non-commercial antibody gave stronger signal, next experiments were carried out with this antibody. This low temperature-induced concentration in the Golgi area was not observed in other enzymes involved in tubulation, such as cytosolic phospholipase A_2_ group IV and lipid phosphate phosphatase 3 (data not shown).

**Figure 3:**
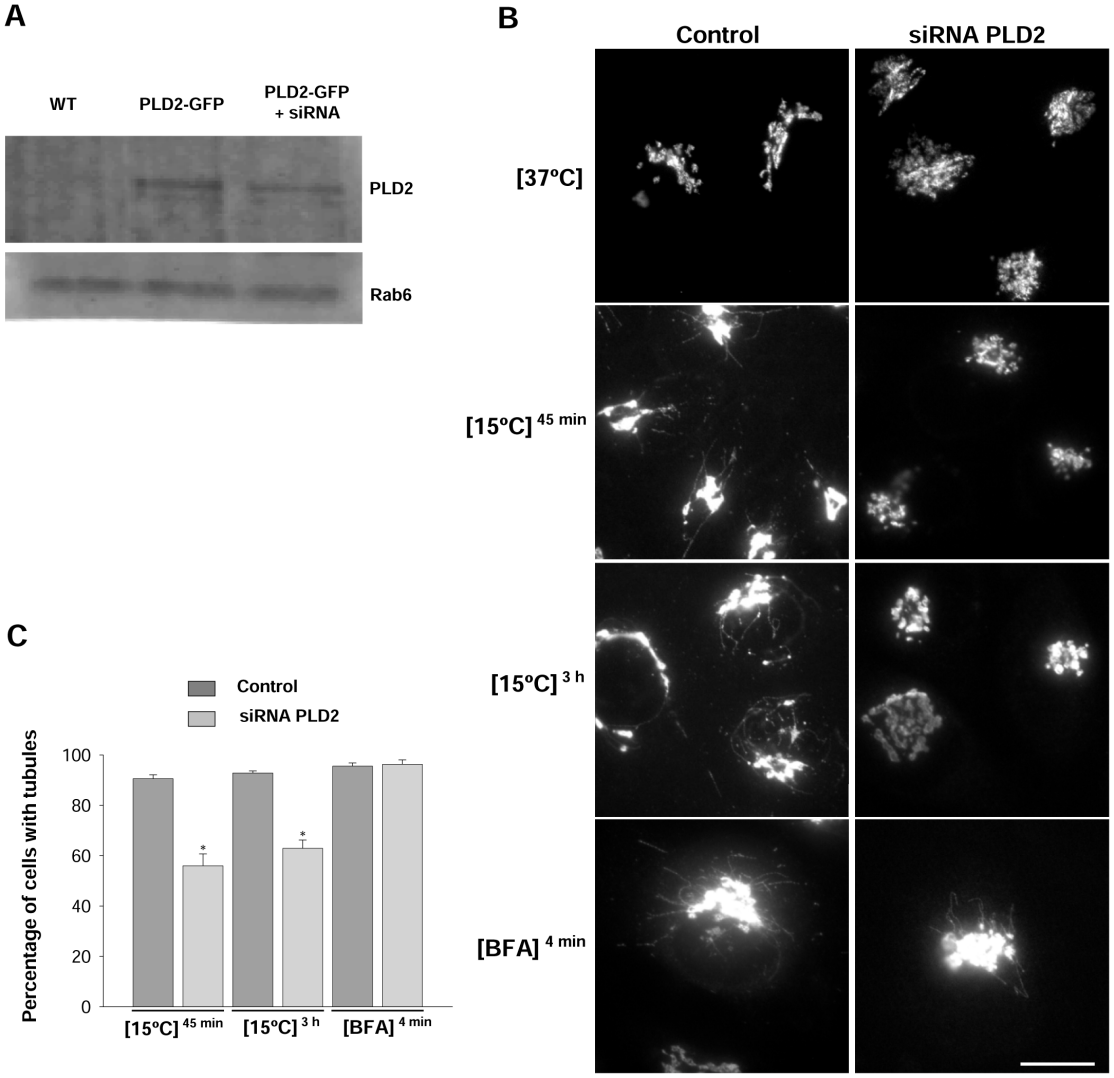
Effects of PLD2 depletion in Golgi tubule formation. (A) Lysates of wild-type cells (WT), cells overexpressing GFP-tagged PLD2 (GFP-PLD2) and cells overexpressing GFP-tagged PLD2 and depleted for PLD2 using siRNA (GFP-PLD2+ siRNA) were immunoblotted using our monoclonal antibody against PLD2 or a polycolonal antibody against Rab6. PLD2 antibody detects a 117 kda band which correspond with the tagged form of the enzyme. Expression of GFP-PLD2 but not Rab6 is effectively decreased by RNA interference. The antibody against PLD2 does not recognize the enzyme in wild-type cells. (B) Control and PLD2-depleted cells were cultured at 15°C (45 min or 3 h) or treated with BFA (4 min) and immulabelled for N-acetyl-galactosaminyltransferase T2. At low temperature, PLD2-depleted cells showed a typical perinuclear labelling pattern but hardly any tubules. However, depletion did not affect tubule formation in BFA experiments. (C) Quantitative analysis. Numbers represent percentage (±SEM) of cells showing tubules (n = 300). Asterisks indicate significant differences (Student t test p≤0.05). Bars, 20 µm.

The present result pointed to PLD2 as the isoform involved in the formation of low temperature-induced Golgi tubules; therefore to confirm this role, we analysed the formation of Golgi tubules in depleted cells by using small interfering RNA (siRNA). Unfortunately, the antibodies used only detected the enzyme in over-expressing cells; thus, so depletion was measured in cells overexpressing GFP-tagged PLD2. The tagged enzyme was downregulated by 30–40% 24 h after transfection with siRNA (Fig. 3A). Moreover, analysis of real-time quantitative PCR demonstrated that endogenous PLD2 expression decreased by 56% 48 h after transfection with siRNA. This drop in the levels of PLD2 affected the formation of this type of tubules (Fig. 3B). Thus, the percentage of cells with Golgi tubules decreased from 80–90% to 55–60% in depleted cells cultured at 15°C for 45 min or 3 h (Fig. 3B). However, depletion affected neither tubule formation (Figs 3B, C) nor Golgi-to-ER redistribution in BFA experiments (data not shown). Taken together, our results show that PLD2 is necessary for the formation of low temperature-induced but not for the BFA-induced tubules, which suggests that there are two types of tubules in the Golgi complex, with different formation mechanisms and, probably, different functions.

### Inhibition of PLD2 activity induces the formation of tubules containing Golgi matrix proteins

**Figure 4:**
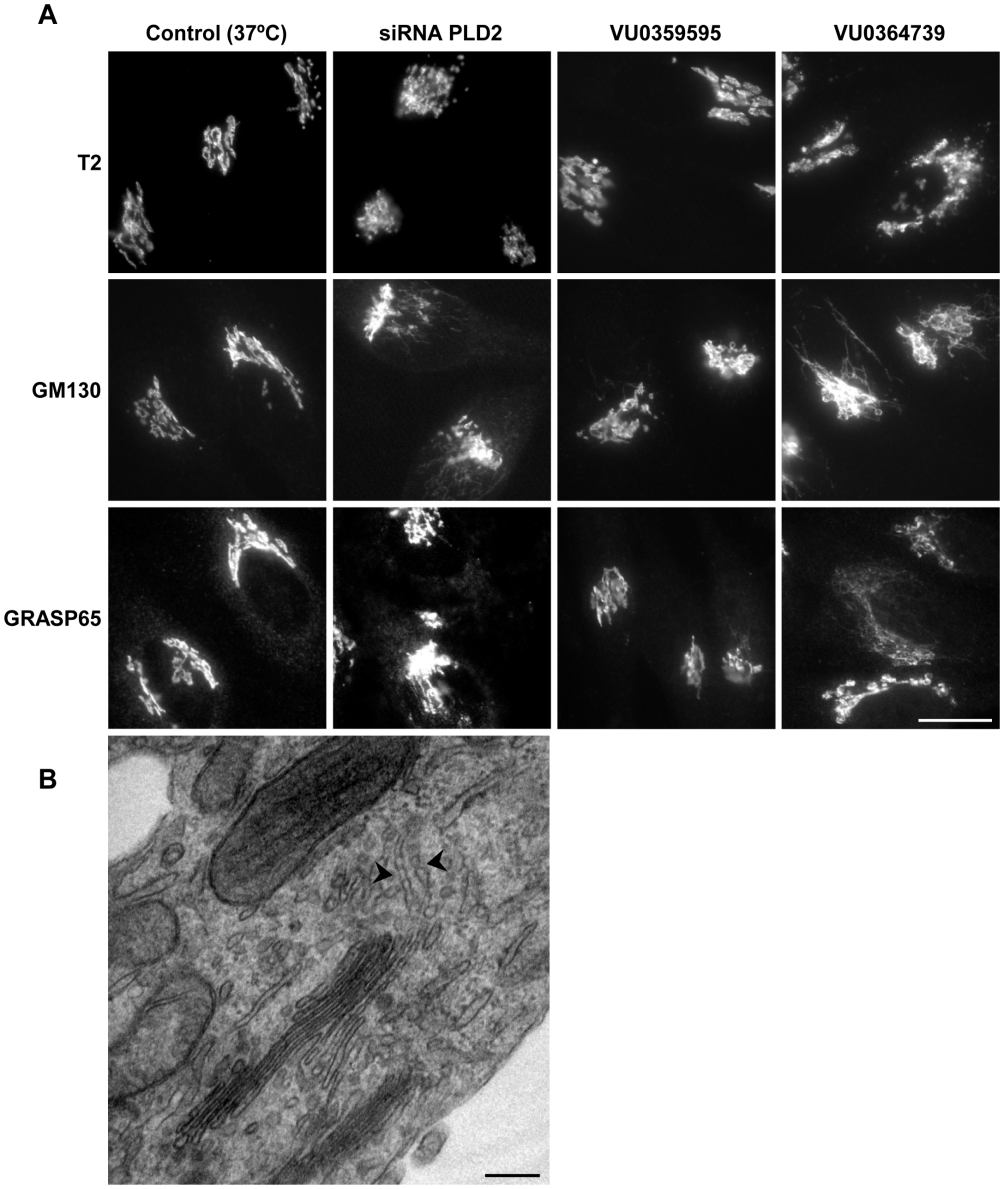
Effect of the depletion and inhibition of PLD2 in the morphology of the Golgi complex. (A) Cells cultured at 37°C were transfected with siRNA against PLD2, treated with VU0359595 (PLD1 inhibitor) or VU0364739 (PLD2 inhibitor) and immunolabelled for N-acetyl-galactosaminyltransferase T2, GM130 or GRASP65. Both siRNA-PLD2 and VU0364739 treatments induced the formation of tubules containing the Golgi matrix proteins GM130 and GRASP65 but not the Golgi resident enzyme T2. (B) Electron microscopy analysis of Golgi complex of PLD2-depleted cells. Arrowheads point to tubules emerging from lateral rims and cis cisternae containing necks (arrowheads). Bars (A), 20 µm; (B), 200 nm.

Low temperature experiments support a role for PLD2 in the formation of Golgi tubules. To confirm this role we explored the role of this enzyme under physiological conditions (37°C) and using different approaches: (i) decreasing its expression level of the enzyme by small interfering RNA (ii) blocking its activity by a specific inhibitor; (iii) over-expressing the enzyme and, finally, (iv) analysing its distribution within the Golgi membranes at ultrastructural level. First, we analysed how PLD2-depletion affected Golgi morphology. As previously described [18], immunofluorescence analysis using a large number of Golgi markers (resident enzymes, Rabs, SNAREs) confirmed that the Golgi morphology of depleted cells showed no major alterations, most of them being of typical perinuclear appearance (Fig. 4A). Only a small percentage of them had a compact round morphology (data not shown). However, we found that PLD2 depletion by siRNA induced the formation of tubules containing the Golgi matrix proteins GM130 and GRASP65 (Fig. 4A). It should be mentioned that this tubulation was not observed in the case of cis Golgi markers such as Rab 1. Electron microscopy confirmed that the architecture of the Golgi complex in PLD2-depleted cells was normal (Fig. 4B). Although tubules are difficult to observe in 2D images, some were observed emerging from the lateral rims and cis cisternae. To confirm that tubulation in depleted cells was specifically related with the enzymatic activity of PLD2 we used the isoform-specific inhibitors VU0364739 and VU0359595 which are 75-fold and 1700-fold selective for PLD2 and PLD1, respectively [33], [34]. The Golgi complex of cells treated with PLD2 inhibitor showed a normal morphology when analysed by immunofluorescence for most Golgi markers (Fig. 4A). However, once again, we observed GM130 and GRASP65-positive tubules (Fig. 4A). In fact, a much greater level of tubulation was observed with this inhibitor than following depletion probably because VU0364739 inhibit PLD2 activity more than depletion does. Conversely, PLD1 inhibitor did not induce tubulation (Fig. 4A).

**Figure 5:**
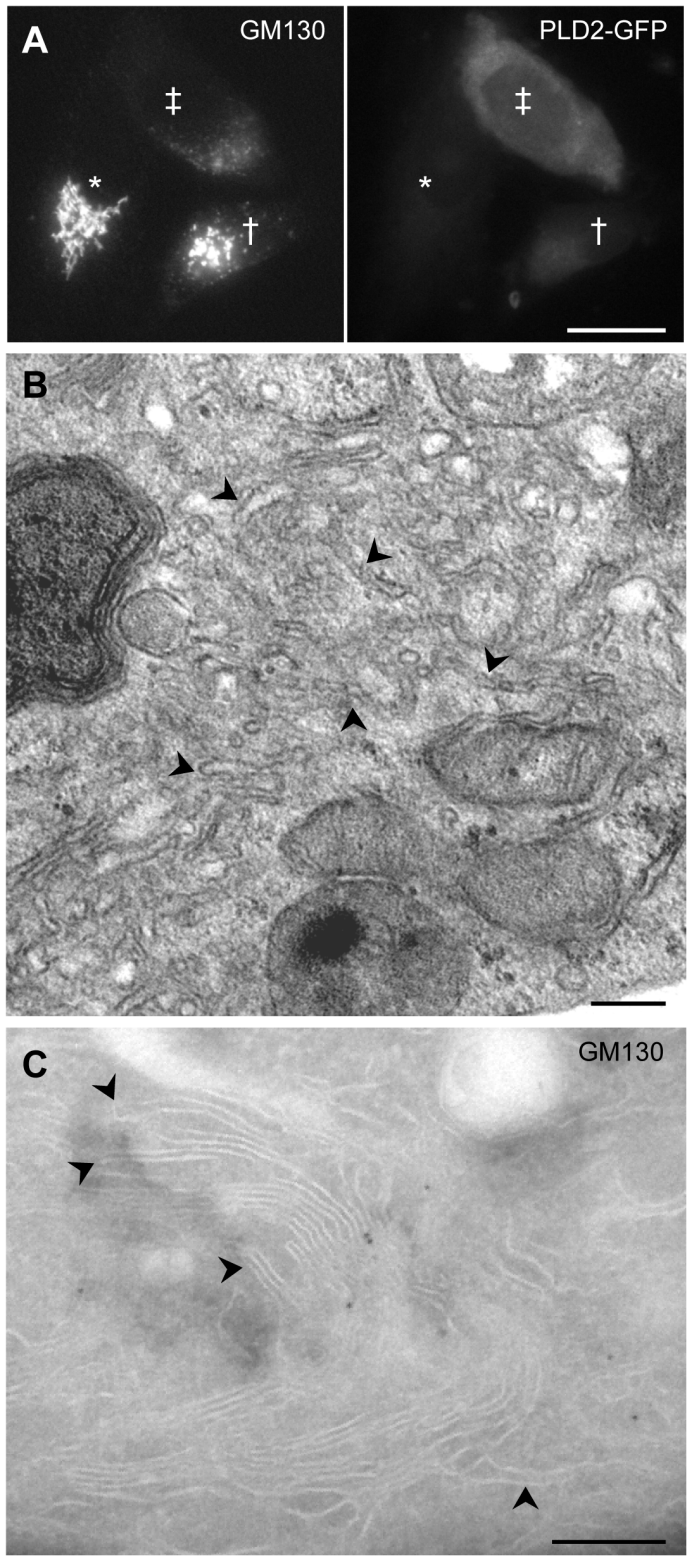
Effect of the over-expression of PLD2 on Golgi morphology. Immunofluorescence (A), electron microscopic (B) and cryoimmunoelectron microscopic (C) analysis of the effect of the over-expression of PLD2 in cells cultured at 37°C. (A) Cells transiently expressing GFP-tagged PLD2 were immunolabelled for GM130. Cells over-expressing high level of PLD2 (‡) apparently lost the Golgi complex. The morphology of this organelle in cells expressing low level of PLD2 (†) is altered in comparison with non-transfected cells (*). (B) PLD2-overexpressed cell showing a highly disorganised Golgi complex formed by tubular networks (arrowheads). (C) Cell expressing GFP-tagged PLD2 was immunolabelled for GM130. The morphology of the Golgi stack is altered and it shows a weak immunoreactivity for GM130. Despite of the difficulty to observe tubules in ultrathin sections, several tubules (arrowheads) emerging from the stack can be identified. Bars (A), 20 µm; (B, C), 200 nm.

We also analysed how the over-expression of PLD2 might affect Golgi morphology. The GFP-tagged form of this isoform showed a similar distribution in the cell to the endogenous enzyme (Fig. 5A). However, to our initial surprise, the Golgi complex of over-expressing cells was apparently lost when analysed by immunofluorescence (Fig. 5A). This loss was not due to protein degradation as confirmed by western blot analysis (Fig. 3A). Electron microscopical analysis of PLD2-overexpressed cells showed a disorganised Golgi complex, where most stacks were replaced by tubular networks (Fig. 5B), supporting that this enzyme regulates Golgi morphology. Cells with low levels of PLD2-GFP showed an altered GC with a high number of tubules (Fig. 5C).

**Figure 6:**
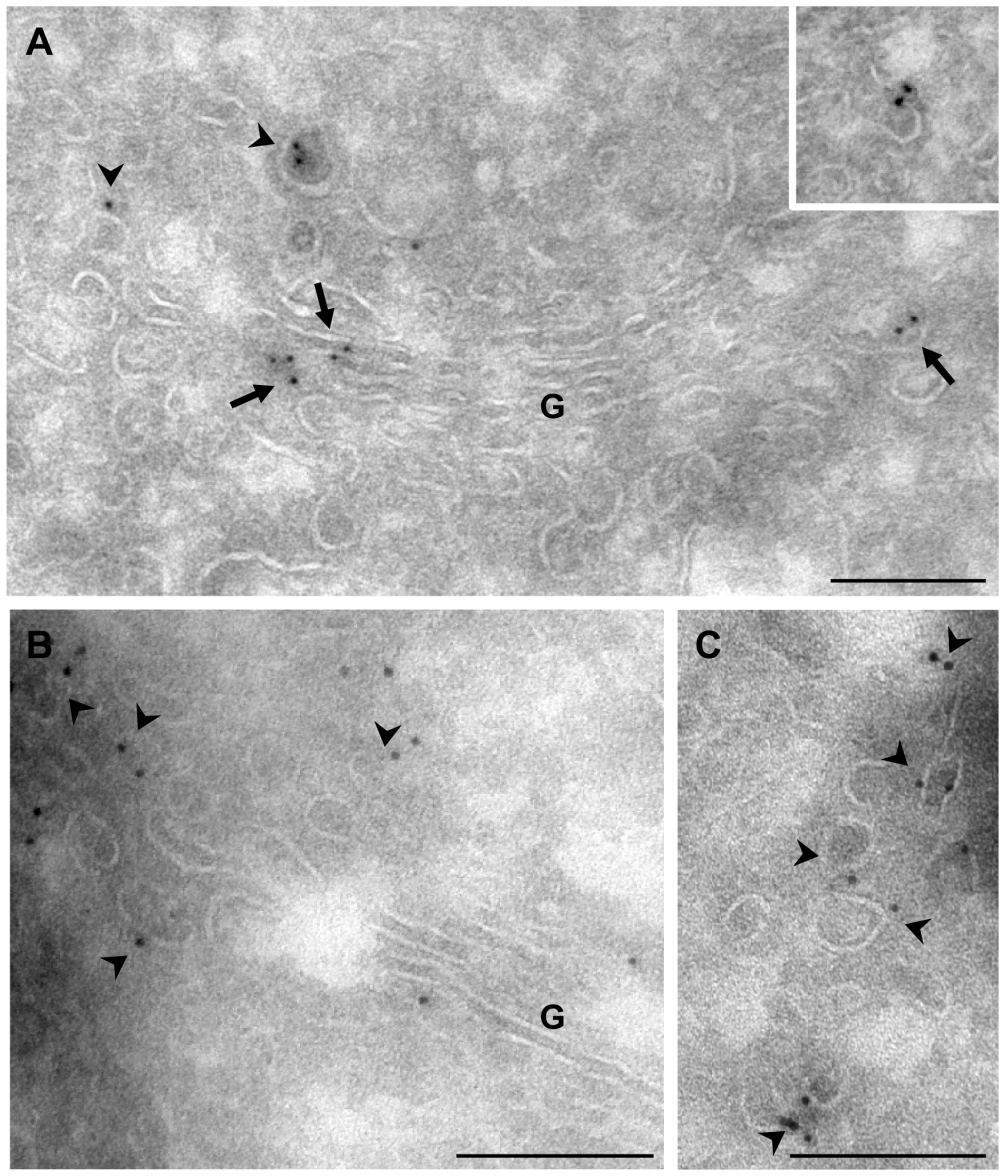
Ultrastructural distribution of PLD2. Ultrathin cryosections of HeLa cells were immunolabelled for PLD2 followed by 10-nm gold-conjugated protein A. (A–C) This enzyme is located on cisternae (G), including their rims (arrows), as well as coated and uncoated peri-Golgi tubule-vesicular membranes (arrowheads). The inset in A shows a COPI-coated bud at the lateral rim of the cisterna. Bars, 200 nm.

Finally, we assessed the localization of PLD2 within the Golgi membranes by cryoimmunoelectron microscopy. A previous study in GH3 cells showed that this enzyme is located in the lateral rims of the Golgi complex, as would be expected if it plays a role in tubulation. In order to analyse these data in HeLa cells and obtain detailed information about this distribution, mild fixation conditions (4% paraformaldehyde) were used. It was found that the enzyme is located on cisternae (61%) and peri-Golgi tubule-vesicular elements (39%) (Fig. 6). PLD2 was observed in lateral zones of the cisterna (Fig. 6A), including COPI-coated buds (inset in Fig. 6A) as well as flat portion of cisternae (Fig. 6B). Most of the immunolabelled vesicles were uncoated (80% of the immunolabelled vesicles) although some showed clathrin (12%) and COPI (7%) coats.

Taken together, our results support the idea that PLD2 is an important player in Golgi tubulation. The present results also provide clues on how different types of tubules may be regulated (see Discussion).

### Association of ArfGAP1 in the Golgi membrane depends of PLD2 activity

**Figure 7:**
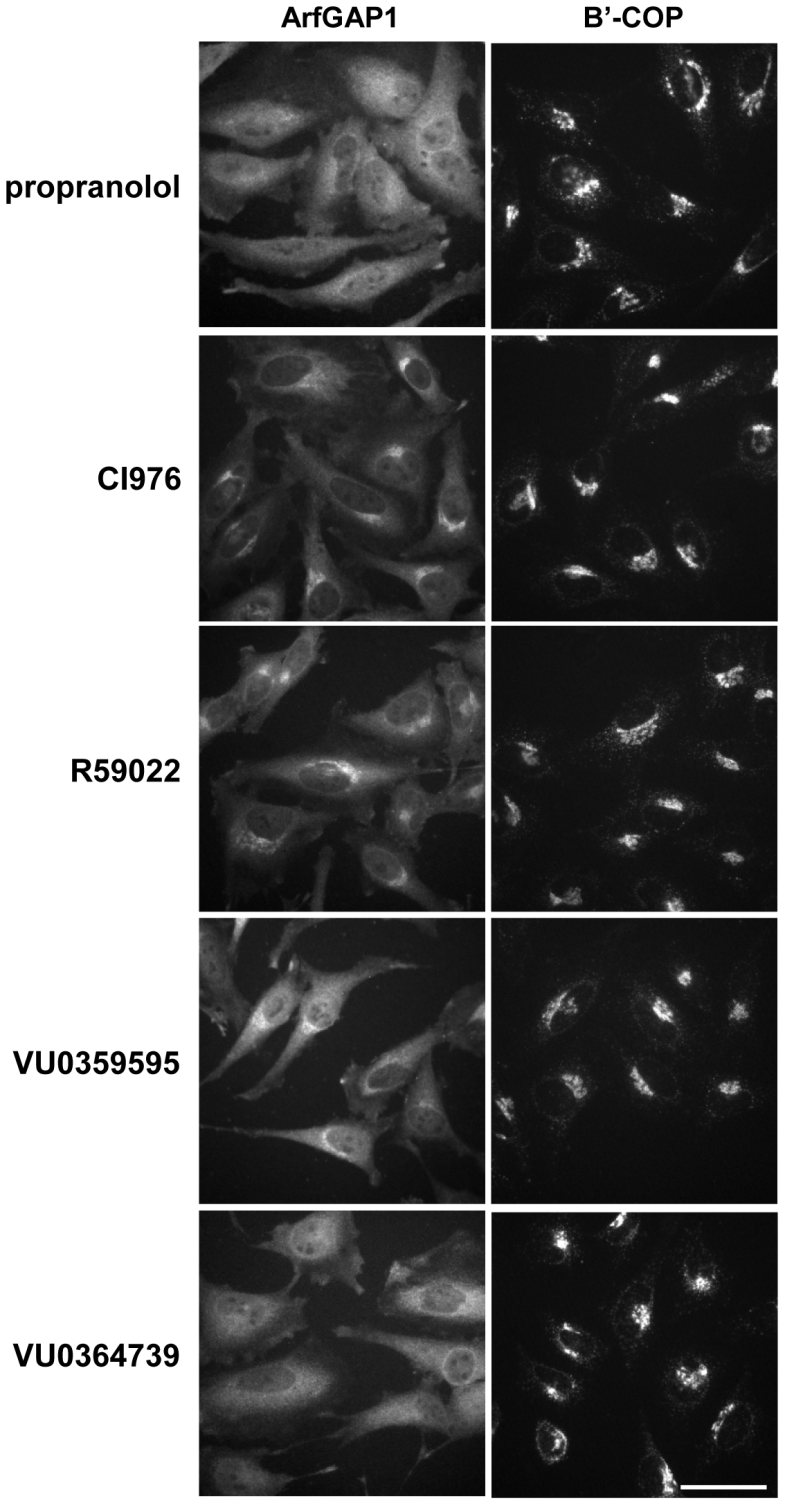
Effects of the alteration of PA levels on the distribution of ArfGAP1 and β′-COP. Cells cultured at 37°C were incubated for 1 h with the corresponding inhibitor and immunolabelled for ArfGAP1 or β′-COP. When cells were treated with the PLD2 inhibitor VU0364739 and propranolol, ArfGAP1 but not β′-COP is redistributed from Golgi membranes. Bars 20 µm.

**Figure 8:**
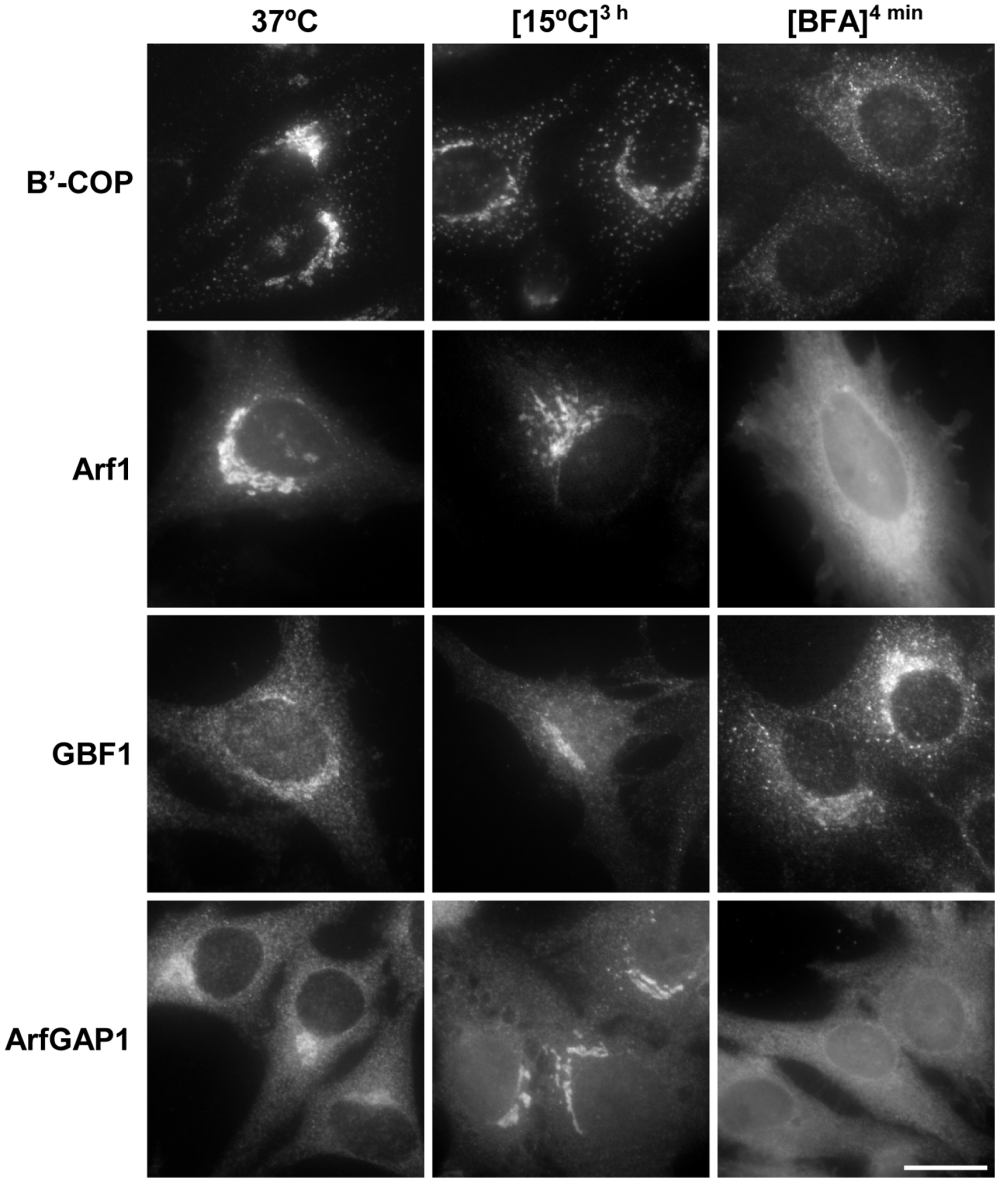
Effect of 15°C and BFA on the distribution of the COPI components. Immunofluorescence analysis of the distribution of COPI, Arf1, GBF1 and ArfGAP1 in control, low temperature (3 h), and BFA (4 min)-treated cells. BFA and low temperature affected components of the COPI machinery differently. Bars 20 µm.

We found that some PLD2 was associated to COPI coats. In addition to a specific lipid composition, Golgi tubulation may require COPI coat machinery. The association of a GTP-tagged form of ArfGAP1 with the Golgi complex depends on DAG [16]. We wanted to know whether there is a relation between the PA generated by PLD2 and the association of ArfGAP1 with Golgi membranes. For this purpose, the way in which lipid inhibitors affected ArfGAP1 distribution was analysed. Endogenous ArfGAP1 was detected using a polyclonal antibody that specifically recognises this protein but not ArfGAP2/3 (data not shown). Of the inhibitors used, only propranolol and the PLD2-specific inhibitor VU0364739 induced ArfGAP1 redistribution from Golgi membranes (Fig. 7). None of these inhibitors affected the Golgi location of other COPI components/regulators, including β′COP (Fig. 7), Arf1 and GBF1. This suggests that ArfGAP1 interacts with DAG and/or the PA generated by PLD2 during the formation of different types of Golgi tubule. To confirm this, we analysed how the association of ArfGAP1 with Golgi membranes was affected during the massive Golgi tubulation induced by low temperatures as opposed to short treatment (4 min) with BFA (Fig. 8). It was found that BFA induced a fall in Golgi-associated ArfGAP1 as described previously [35], whereas low temperature induced the opposite effect - its recruitment into the perinuclear area. This recruitment was quantitatively analyzed by measuring the immunofluorescence signal in the Golgi area in relation to the rest of the cytoplasm. We found that the immunofluorescence in the Golgi area significantly increased 13% and 27% after 45 min and 3 h of incubation at low temperature, respectively (Student's test, p<0.05). Although both BFA and low temperature induce the detachment of coatomer proteins [6], they affected other components of the COPI machinery differently (Fig. 8). Thus, Arf1 remained in the Golgi area, including tubules, after exposure to low temperature, while BFA rapidly expelled it from the Golgi membranes. Low temperature did not affect the distribution of GBF1, the GEF for Arf1, whereas it concentrated in the Golgi area after BFA addition. COPI-induced membrane deformation also depends on BARS [18], a process which depends on the PA generated by PLD2. However, neither BFA nor low temperature affected the distribution of BARS (data not shown). Together, these results support the idea that the association of ArfGAP1 to the Golgi membranes is regulated by PLD2 activity and suggest that ArfGAP1 may play a role in the formation of a specific subset of tubules.

## Discussion

PLD is associated with important cell signalling pathways that regulate cell survival, proliferation, migration, cytoskeletal organization, endocytosis and exocytosis, differentiation and defence [23], [30], [36]. The altered expression of PLD has been associated with several diseases, including cancer and neurodegeneration [37]. The product of the enzyme, PA, is an anionic phospholipid which is able to recruit and activate positively charged proteins. In addition, it can be converted into other signalling molecules such LPA and DAG [38]. So far, PLD has been found to interact with 58 proteins and 5 lipids [36]. In mammals, there are two major isoforms, PLD1 and PLD2, which are widely expressed [39] and share 50% homology [40]. PA generated by PLD2 has been seen to be involved in the late steps of COPI-coated vesicle formation, more specifically in the scission of budding vesicles [18]. PLD2 inhibition affects COPI retrograde transport but not anterograde intra-Golgi transport [19]. However, in contrast with our results, these studies failed to find a role for this enzyme in membrane tubule formation. Here, we show that this enzyme and its product, PA, are key regulators in Golgi tubule formation. We first demonstrate that low temperature-induced tubules, in contrast to their BFA-induced counterparts, require PA. The data obtained with different inhibitors suggest that all routes that regulate the amount of this lipid contribute to tubule formation. The fact that DAG and LPA are also involved in tubule formation [41] led us to think that they might regulate the formation of different types of tubules. However, it might also be possible that tubulation is an unspecific attribute of Golgi membranes, which may be very sensitive to the lipid composition, and any slight change in their composition may artificially alter their curvature. However, the specific composition of the tubules induced by low temperature and PLD2 depletion argue against this idea.

PA can be generated by the action of LPA acyltranferase, DAG kinase and PLD. In our low temperature experiments, the maximum inhibition was obtained with 1-butanol, therefore we focused on PA generation from phosphatidycholine catalysed by PLD. Low temperatures induce a redistribution of PLD2 isoform from cytoplasm to the Golgi area. It should be mentioned that mammalian PLD maintains high enzymatic activity at temperatures around 15°C [42]. The fact that low temperatures induce movement of peripheral ERGIC elements to the Golgi area [43] may explain the increased labelling observed at the perinuclear area. We found some PLD2-immunoreactivity in ERGIC elements. Our EM images, under physiological conditions, showed PLD2-immunolabelled peri-Golgi tubulovesicular elements, which may correspond to these intermediates. It might be also possible that low temperature affect the recycling of the enzyme thorough ERGIC/Golgi membranes. Conversely, the increase in perinuclear labelling might correspond to a pool of cytosolic enzyme recruited to the Golgi membranes. PLD2 (and PLD1) have PX and PH domains with an affinity for phosphoinositidies, but low affinity for Golgi-associated phosphoinositidies [44]. Its specific binding to membranes and the intracellular location of PLD2 also depends on palmitoylation in the PH domain [44]. In fact, the exact distribution of the isoforms is not well known, but PLD2 seems to be more membrane-bound, including lipid rafts [36], in contrast to PLD1, which is more cytosolic when expressed in insect cells [23].

The role of PLD2 in tubule formation was also confirmed under physiological conditions. The endogenous enzyme is located in the lateral rims of the Golgi apparatus from which the tubules grow (this study and [32]). Second and importantly, depletion experiments and the use of specific inhibitors confirmed that this enzyme is involved in the formation of a specific set of Golgi tubules containing matrix proteins. Finally, over-expression of a GFP-tagged form of enzyme induced the replacement of stacked cisternae by tubular networks. The final conclusion is that this isoform is a key regulator of Golgi morphology and tubule formation. Activation or inhibition of the enzyme will induce the formation of specific tubules containing resident enzymes or matrix proteins, respectively. The different results obtained with or without pre-treatment suggest that PA is important in an early step of tubule formation. Conversely, our depletion studies support a role for this enzyme in the fission of a specific set of transport carriers. Thus, as described for DAG [15], [16], this lipid may be involved in different steps of carrier biogenesis.

Apart from their specific lipid composition, coat complexes may be involved in tubule formation. We found that low temperature and BFA affect COPI coat components and regulatory molecules very differently. Although low temperature also induces the detachment of COPI coat proteins, the process is very slow compared with BFA [6]. The most dramatic and interesting difference between BFA and low temperature treatments is found in the case of ArfGAP1, which is recruited in the Golgi area at 15°C but detached from these membranes soon after the addition of the fungal drug. The specific recruitment of ArfGAP1 into the Golgi area at low temperature suggests that this phenomenon might be related to tubule formation. The relationship between COPI coats and lipid composition during transport carrier formation has recently been demonstrated. The coordinated action of LPA acyltransferase type γ and cytosolic phospholipase A_2_ type α acting on COPI coats facilitates the formation of vesicles or tubules, respectively [19]. Moreover, Arf1 stimulates PLD activity, although to a greater extent in the case of PLD1 isoform [23]. Our EM images support that PLD2 is associated with COPI coats, confirming previous results on the role of this isoform in retrograde transport. Importantly, experiments based on specific lipid inhibitors suggest that PA specifically generated by PLD2 is necessary for the recruitment of ArfGAP1 to membranes. However, ArfGAP1 remained in the Golgi area in PLD2-depleted cells (data not shown) probably because the diminution of PA in these silenced cells (∼60%) is not as great as in cells treated with 10 µm of inhibitor (100%) [34]. DAG also seems to be necessary for this recruitment (our results and [16]). It is possible that both lipids are needed simultaneously for this process, as described previously for other proteins [29]. The role of ArfGAP1 is controversial [45], [46]. Classically, it was considered that the hydrolysis of GTP in GTP-bound Arf triggers the loss of the COPI coats. Conversely, it might be involved in coat polymerization, promoting vesicle formation. Its role in cargo sorting is also under discussion. PA interacts with members of the GAP family, including AGAP1 [47] and Rho-GAP [48], although, to the best of our knowledge, such interaction has not been described for ArfGAP1. ArfGAP1 binds membranes containing lipids with spontaneous negative curvature because of its ability to sense packing defects of the membrane, for instance, when conical shaped lipids are incorporated into membranes made of cylindrical lipids [49]. DAG and PA have conical forms and induce spontaneous negative curvature [50]. ArfGAP1 binds highly curved membranes, although this is not the case with BFA-treated cells. The interaction between PA and ArfGAP1 could be indirect (through the protein BARS) [18] although this is unlikely because this protein, in contrast to ArfGAP1, was not recruited into Golgi membranes in our tubulation models (data not shown). It is interesting that PLD1-generated PA is not involved in the binding of ArfGAP1 to Golgi membranes, as was demonstrated by the use of a specific inhibitor. *In vitro* analysis showed that both isoforms elaborate the same PA species [51]. However, this may not be the case *in vivo* due to the specific lipidic environment of cellular membranes.

Our results suggest that Golgi membranes are able to generate different types of tubules of varied composition, as it has been postulated for COPI vesicles [52]. Lipid-generating enzymes such as LPA acyltransferase, phospholipase A, DAG kinase and LPP are key regulators of this process. PLD2 must be included in this list. When PLD2 enzymatic activity is enhanced or the enzyme is recruited to certain Golgi areas, the PA generated is able to bend the membrane and to recruit ArfGAP1, which might be necessary for the recruitment of resident enzymes [53]. Conversely, when its activity is inhibited, there is an accumulation of DAG or LPA, which might generate another type of tubule containing matrix proteins. It can be speculated that some Golgi tubules may be involved in retrograde intra-Golgi traffic [6], [54], whereas others keep the architecture of the Golgi ribbon [54] or anterograde intra-Golgi transport [55]. Thus, new experiments are needed to shed light on the fine tuning of tubular regulation.

## Materials and Methods

### Antibodies and reagents

A monoclonal antibody against PLD2 was generated by Abmart (Shanghai, China) using the KEGEDPADRM epitope sequence of the human protein. Polyclonal antibodies against PC-PLD1 and PC-PLD2 were purchased from Santa Cruz Biotechnology (Santa Cruz, CA, USA). Rabbit polyclonal antibodies against ArfGAP1 and GBF1 were obtained from Sigma-Aldrich (Madrid, Spain). Rabbit polyclonal against GRASP65 was purchased from Abcam (Cambridge, UK). The source of other primary antibodies has been previously described [6], [7]. Secondary mouse and rabbit antibodies coupled to Alexa Conjugated (488, 568) were obtained from Invitrogen (Barcelona, Spain). Protein A-gold was obtained from the department of Cell Biology at Utrecht University (Utrecht, The Netherlands). The plasmids coding PLD2-GFP and Arf1-GFP were obtained from OriGene (Rockville, MD, USA). The small interfering RNA (siRNA) for PC-PLD2 was obtained from Santa Cruz Biotechnology.

BFA, 1-butanol, propranolol, 6-{2-{4-[(p-Fluorophenyl)phenylmethylene]-1-piperidinyl}ethyl}-7-methyl-5H-thiazolo(3,2-a)pyrimidine-5-one (R59022) and 2,2-Dimethyl-N-(2,4,6-trimethoxyphe¬nyl)dodecanamide (CI976) and unspecified chemical reagents were purchased from Sigma. (1R, 2R)-N-([S]-1-{4-[5-bromo-2-oxo-2,3-dihydro-1H-benzo(d)imidazol-1-yl]piperidin-1-yl}propan-2-yl)-2-phenylcyclopropanecarboxamide (VU0359595), a selective phospholipase D1 inhibitor, was obtained from Avanti Polar Lipids (Alabaster, AL, USA). N-[2-[1-(3-Fluorophenyl)-4-oxo-1,3,8-triazaspiro[4.5]dec-8-yl]ethyl]-2-naphthalenecar boxamid (VU0364739), a selective phospholipase D2 inhibitor, was purchased from Tocris Bioscience (Bristol, UK). A frozen stock solution of 20 mM CI976 and 2 mM R59022 were prepared in DMSO. VU0353595 and VU0364739 were prepared as a 10 nM stock solution in DMSO and stored at 4°C.

### Cell culture and experimental treatments

HeLa cells (ATTC CRM-CCL-2) were cultured in DMEM (Gibco, Paisley, UK) containing 10% FBS under standard tissue culture conditions (37°C, 5% CO2). To induce Golgi tubule formation, cells were cultured in a temperature-controlled incubator at 15°C for different periods of time (45 min or 3 h) or, conversely, cells were treated with freshly prepared BFA (1 µg/mL) in DMEM for 4 minutes at 37°C. Cells were grown on glass coverslips and incubated with freshly prepared inhibitors before each experiment. The following concentrations of enzyme inhibitors were used: 0.3% 1-butanol, 60 µM propranolol [16], 20 µM CI976 [27], 20 µM R59022 [29], 10 µM VU0359595 [33] or 10 µM VU0364739 [34].

### Transfection and protein depletion

HeLa cells were transiently transfected with the plasmids using Lipofectamine 2000 (Invitrogen) according to the manufacturer's instructions. After 18 hours of protein expression, cells were fixed and processed for immufluorescence or electron microscopy.

PLD2 downregulation by means of the siRNA approach was performed according to the manufacture’s protocol (Santa Cruz Biotechnology). The knockdown efficiency after 48 h was determined by RT-PCRrt, using GAPDH mRNA to normalize RNA inputs. Efficiency was also determined by SDS–PAGE and subsequent immunoblot analysis of cells overexpressing GFP-tagged-PLD2 and depleted for 24 h.

### Immunofluorescence microscopy

Cells grown on glass coverslips to 70% confluence were fixed with 4% paraformaldehyde in PBS for 10 min at room temperature. After washing with PBS, blocking with PBS-glycine and permeabilization with 0.1% saponin, the samples were processed for indirect immunofluorescence, as described previously [6], [7]. The coverslips were examined with a Zeiss Axiophot fluorescent microscope coupled to a Leica DC 500 digital camera.

Quantitation of the percentage of cells immunolabelled for N-acetyl-galactosaminyltransferase T2 showing at least one unambiguously identified Golgi tubule was performed by analysing 300 cells per treatment (Figs 1I and 3). Only tubules unambiguously connected to the Golgi complex (perinuclear area) were used to analyse the mean number of tubules per cell (Fig. 1J).

### Ultrastructural analysis

For conventional electron microscopy, cells were fixed with 2% glutaraldehyde in 0.2 M cacodylate buffer, pH 7.4 for 120 min at 4°C, post-fixed for 1 h in a mixture 1∶1 of 2% OsO4 and 3% of potassium ferrocyanide, dehydrated and embedded in Epon 812. For cryoimmunoelectron microscopy, cells cultured at 37°C were fixed with 4% paraformaldehyde in 0.1 M sodium phosphate buffer, pH 7.4 and processed as described previously [6], [7]. Grids were examined with a Jeol-1011 transmission electron microscope. For quantitative analysis, 15 Golgi complexes immunolabelled for PLD2 were randomly selected. In the Golgi area, gold particles were counted and ascribed to one of the following categories: lateral portion of cisterna (defined as the lateral zones of stacked cisternae that show the characteristic terminal dilatation), central portion of cisterna (defined as the flattened zones of stacked cisternae), and peri-Golgi tubule-vesicular elements (defined as membrane profiles located in the vicinity of the Golgi stacks). Clathrin and COP coats were identified by their characteristic 18 and 10 nm thick coats, respectively.
